# A case of topical 5-fluorouracil provoked psoriasis

**DOI:** 10.1016/j.jdcr.2023.06.036

**Published:** 2023-07-06

**Authors:** Kevin S. Puerta Durango, Chiamaka L. Okorie, Shabnam Momtahen, Brian J. Simmons

**Affiliations:** a Geisel School of Medicine at Dartmouth College, Lebanon, New Hampshire; b Department of Pathology and Laboratory Medicine, Dartmouth Hitchcock Medical Center, Lebanon, New Hampshire; c Department of Dermatology, Dartmouth Hitchcock Medical Center, Lebanon, New Hampshire

**Keywords:** 5-fluorouracil, cutaneous drug reaction, drug-induced, drug-provoked, psoriasis

## Introduction

Drug-induced psoriasis has been traditionally reported with medications such as beta-blockers, lithium, and anti-malarial drugs, but reports of 5- fluorouracil (5-FU) causing psoriasis are sparse.^1^ 5-FU is an antineoplastic antimetabolite widely used to treat squamous cell carcinoma in situ. In addition, 5-FU also treats actinic keratosis and certain basal cell carcinomas.^2^ 5-FU is known to cause erythema, pruritus, dermatitis, and burning.^3^ However, it is not currently associated with inducing or exacerbating psoriasis. Herein, we present a case of psoriasis arising 2 weeks after applying 5% 5-FU with complete resolution on 5-FU discontinuation.

## Case report

**Fig 1:**
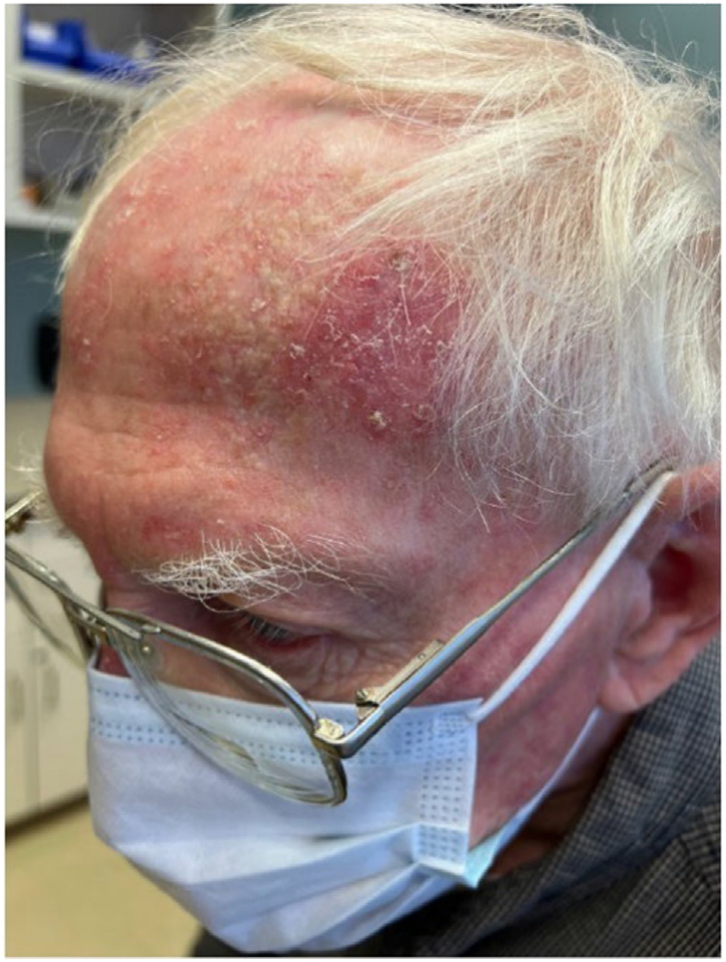
Squamous cell carcinoma in situ – *Pink* scaly plaque on the *left* frontal hairline.

An 82-year-old man with a past medical history of hypertension, hyperlipidemia, COPD, and beta-blocker-induced psoriasis, presented to the clinic with a pink scaly plaque on the left frontal hairline that was concerning for squamous cell carcinoma (Fig 1). His medications included lisinopril, simvastatin, and amlodipine. A shave biopsy showed a squamous cell carcinoma, at least in situ, suspicious for superficial invasion. The patient was started on 5% 5-FU cream twice daily for 4 weeks.

**Fig 2:**
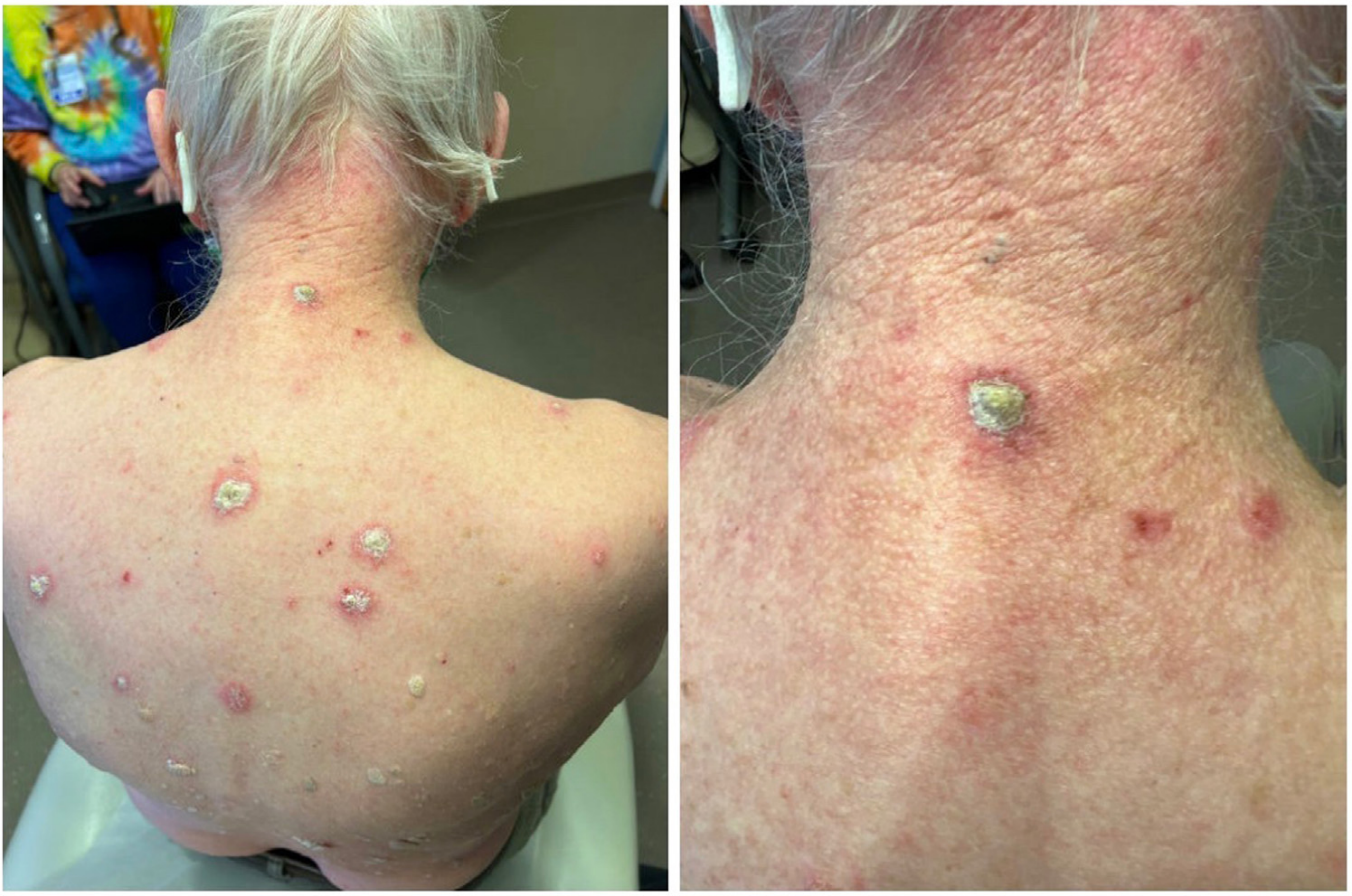
De novo psoriasis - Multiple thick, scaly papules and plaques on an erythematous base on the posterior midline neck.

**Fig 3:**
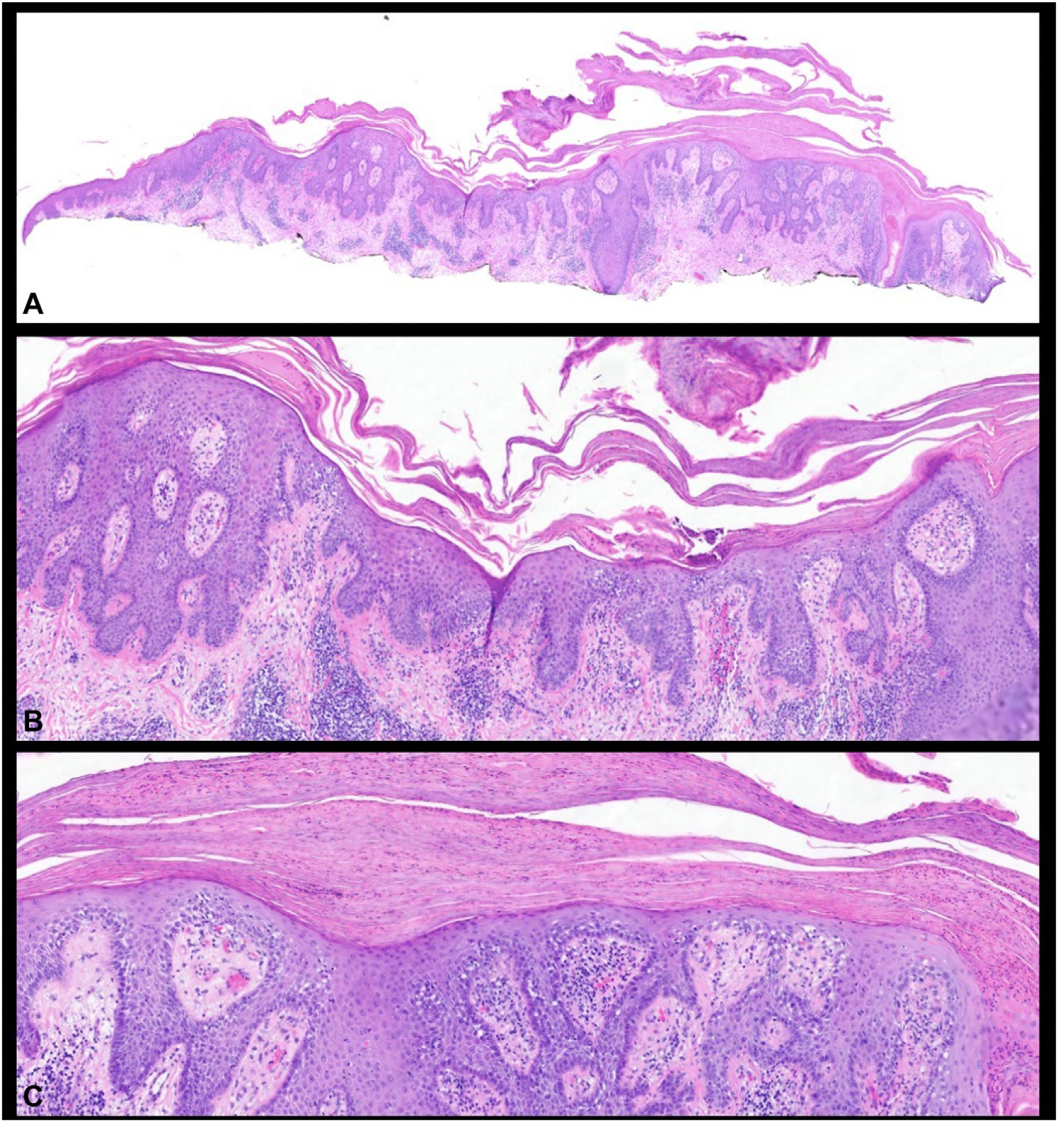
Histopathology of de novo psoriasis - Verrucoid and psoriasiform epidermal hyperplasia, broad parakeratosis, intracorneal neutrophils, loss of granular layer, and elongated rete ridges (**A**. 2×, **B**. 10×, **C**. 20×).

**Fig 4:**
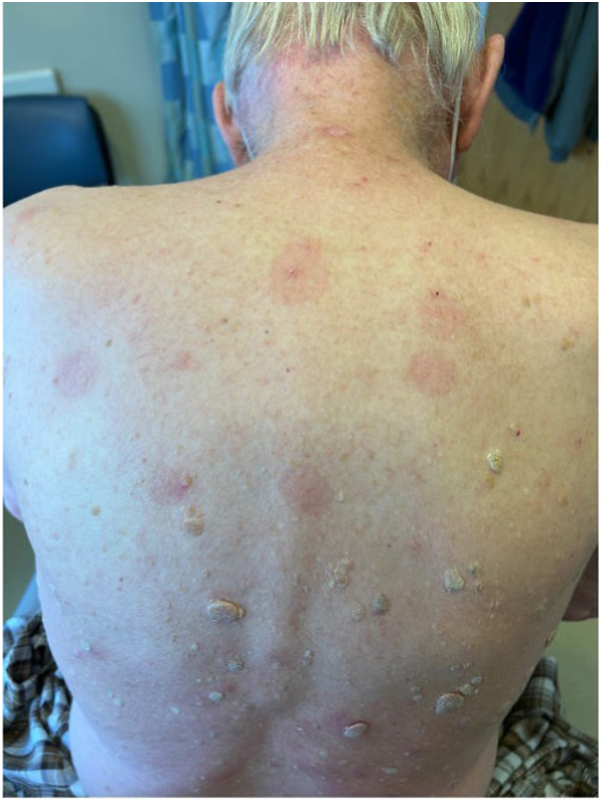
Psoriasis resolution - *Pink* patches on the back in the areas of prior involvement.

After 2 weeks of treatment, the patient returned to the clinic with multiple thick, scaly papules and plaques on an erythematous base on the posterior midline neck (Fig 2) that were becoming irritated and itchy with 5-FU treatment. A shave biopsy showed findings consistent with psoriasis including verrucoid and psoriasiform epidermal hyperplasia, broad parakeratosis, intracorneal neutrophils, loss of granular layer, and elongated rete ridges (Fig 3). There were also superimposed actinic keratosis-type changes. The patient had no current history of trauma, infection, or endocrine disorder. He also denied known psoriasis triggers such as smoking, alcohol consumption, or acute withdrawal of systemic or potent topical corticosteroids. 5-FU was immediately stopped, and no other treatment was advised. At a follow-up visit 1 month later, the exam revealed pink patches on the back in the areas of prior involvement that appeared to be resolving with the discontinuation of 5-FU (Fig 4). Two months later, faint pink patches were noticed, achieving resolution.

## Discussion

Squamous cell carcinoma in situ (SCCIS) presents as an erythematous scaly plaque characterized by intraepidermal dysplasia. Due to the possibility of progression to invasive carcinoma, treatment must be effective at limiting progression to a more aggressive form of cancer. Currently, there is no clear consensus for the best treatment of SCCIS, with different guidelines providing varying levels of support for certain treatments.^4^ Many factors impact the treatment choice, including the patient’s desire for cosmetic results, cost, and ease of application. After considering the various options, 5-FU was an appropriate treatment for this patient.

5-FU is a pyrimidine antimetabolite that impedes DNA synthesis. It prevents cell proliferation and leads to cell death. It has been used effectively for treating many dermatological conditions, including actinic keratosis, superficial basal cell carcinoma, and SCCIS.^2^ Dermatological reactions are the most common side effects of 5-FU. Reported adverse events have focused on erythema, scaling, dryness, and stinging. Systemic side effects such as leukopenia, mucocutaneous ulcerations, and disruption of hair growth often occur in the setting of systemic therapy and are uncommon with topical application because of the limited systemic absorption, as only approximately 6% is absorbed systemically. A different report found that <2% is absorbed systemically in normal skin, systemic absorption may have been as much as 75 times greater in diseased skin.^5^ This potential for a large increase brings up another possible cause of the psoriasis flare being the application of 5-FU over a large surface area increasing the systemic absorption and triggering the psoriasis.

The patient did not recall any history of psoriasis. Still, 1 note in his chart from 11 years ago states a potential beta blocker-induced psoriasis. It is not known how soon after initiating the beta blocker, psoriasis was appreciated, but the suspected beta-blocker was discontinued at the time. Since then, other medications have been used for hypertension management. The mechanism behind beta blocker-induced psoriasis is well-reported. It is believed that this is most likely connected to a decrease in the intra-epidermal cAMP resulting in increased epidermal cell turnover.^6^ Given that our patient has a possible prior history of drug-induced psoriasis, he may have an increased susceptibility to psoriasis inducement by a variety of medications. It is also possible that 5FU may have exacerbated this patient’s psoriasis, which may have had undetected subclinical signs prior to treatment, instead of de novo inducement.

Several drugs have been implicated in provoking psoriasis.^1^ However, there are no reported incidences of 5-FU triggering psoriasis. It is difficult to differentiate between de novo psoriasis and drug-induced psoriasis clinically and histopathologically. On histology, they both present with parakeratosis, psoriasiform hyperplasia, and elongation of the rete ridges.^7^ The onset of psoriasis 2 weeks after the initiation of 5-FU and the subsequent regression and resolution of psoriasis after discontinuation of 5-FU, with no additional triggering factors, provide strong support for a causal relationship between 5-FU and this adverse reaction. Although there is evidence for a relationship between 5-FU and the adverse reaction, it is possible that this was not a direct effect but rather an indirect impact of stress from the application of 5-FU. Unlike beta blockers, which impact the levels of cAMP, it is our theory that the buildup of nucleotide imbalances and DNA misincorporation because of 5-FU causes an increased demand for DNA repair, leading to increased DNA breaks and epidermal cell turnover, ultimately leading to increased susceptibility to psoriasis.^8^ There are limited reports of topical treatment induced psoriasis, but examples like topically applied imiquimod stimulating the production of proinflammatory cytokines and chemokines leading to the development of psoriasis-like skin lesions in mice, gives precedence to topical medications leading to a psoriasis flare.^9^

Reporting cases of possible drug-provoked psoriasis help in the appreciation of the disease process. Our report highlights the importance of close clinical observations while prescribing treatments, even with medications that have well-documented adverse effects as novel reactions can arise. Although 5-FU-induced psoriasis has not been previously reported, this case provides evidence for a causal relationship. Management of all suspected drug-provoked psoriasis such as this must include a thorough history and documentation of the exact timing of initiation of the drug, the dose regimen, and the duration of therapy. Clinicians should also consider other aggravating factors, the relationship between the clinical onset of symptoms and initiation of the new drug, and the improvement of clinical symptoms with discontinuation of the drug when making the diagnosis.^10^ Additionally, prior drug-induced psoriasis should raise caution for predisposition. As such, clinicians should closely monitor for conspicuous signs of psoriasis in patients with a history of drug-provoked psoriasis prior to initiation of new treatment.

## Conflicts of interest

None disclosed.
